# Supplementary material for: Investigating Aerobic Hive Microflora: Role of Surface Microbiome of Apis Mellifera
Source: Biology (Basel). 2025 Jan 17;14(1):88. doi: 10.3390/biology14010088 (PMC11760457; doi:10.3390/biology14010088)
Supplement: Supplementary file 1 [file biology-14-00088-s001.zip › biology-3392538-supplementary.pdf]

Supplementary for:

Investigating aerobic hive microflora: role of surface microbiome of *Apis mellifera*

Kaschenko Grigoriy[1,2], Taldaev Amir[3,4] , Adonin Leonid[3,5*] and Smutin Daniil[5,6*]

[1] - Faculty of geology, soil science and landscape studies, Russian State Agrarian University - Moscow Timiryazev Agricultural Academy, 127550 Moscow, Russia;
[2] - Dokuchaev Soil Institute, 119017 Moscow, Russia;
[3] - Institute of Biomedical Chemistry, 119121 Moscow, Russia;
[4] - Research Center for Molecular Mechanisms of Aging and Age-Related Diseases, Moscow Center for Advanced Studies, 123592 Moscow, Russia;
[5] - Federal State Budget-Financed Educational Institution of Higher Education The Bonch-Bruevich Saint-Petersburg State University of Telecommunications, 193232 St.-Petersburg, Russia;
[6] - Faculty of Information Technology and Programming, ITMO University, 197101 St.-Petersburg, Russia.

[*] Corresponding authors: dvsmutin@gmail.com, leo.adonin@gmail.com.

Supplementary table S1. Composition of the nutrient mediums used in experiments.

Agar	pH	Components	Added g / l
Nutrient 1 "GRM" (Chimmed)	7.3 ± 0.2	Pancreatic fish meal hydrolysate (Chimmed)	12.0
		Peptone dry fermentative (Chimmed)	12.0
		NaCl (Chimmed)	6.0
Nutrient 2 "GMF" (Chimmed)	7.3 ± 0.2	"GMF"-base (MERCK)	15.0
		NaCl (Chimmed)	6.0
Chzapek (MERCK)	7.3 ± 0.2	Saccharose (Lenreaktiv)	30.0
		$NaNO_3$ (Lenreaktiv)	2.0
		K_2HPO_4 (Lenreaktiv)	1.0
		$MgSO_4$ (Chimmed)	0.5
		KCl (Chimmed)	0.5
		$FeCl_3$ (Chimmed)	0.01
Saburo (Chimmed)	5.5 ± 0.2	Papaic digest of soyabean meal (Chimmed)	3.0
		Peptone dry fermentative (Chimmed)	7.0
		Autolyzed yeast extract clarified (Chimmed)	4.0
		Glucose (Sigma-Aldrich)	40.0
SCD (Sigma-Aldrich)	7.3 ± 0.2	Papaic digest of soyabean meal (Chimmed)	3.0
		Casein enzymic hydrolysate (Chimmed)	17.0
		Dextrose (Chimmed)	2.5

		NaCl (Chimmed)	5.0
		K_2HPO_4 (Chimmed)	2.5
A4	7.3 ± 0.2	Papaic digest of soyabean meal (CONDA Pronadisa)	10.0
		Glucose (Chimmed)	10.0
		$CaCO_3$ (Chimmed)	2.5
		NaCl (Chimmed)	5.0
Gauze II	7.3 ± 0.2	Tripton (Chimmed)	3.0
		Peptone (Chimmed)	5.0
		Glucose (Chimmed)	10.0
		$CaCO_3$ (Chimmed)	2.5
		NaCl (Chimmed)	5.0
Oatmeal Agar (solid) (Sigma-Aldrich)	7.3 ± 0.2	Oatmeal (MERCK)	20.0
		Agar (Sigma-Aldrich)	18.0

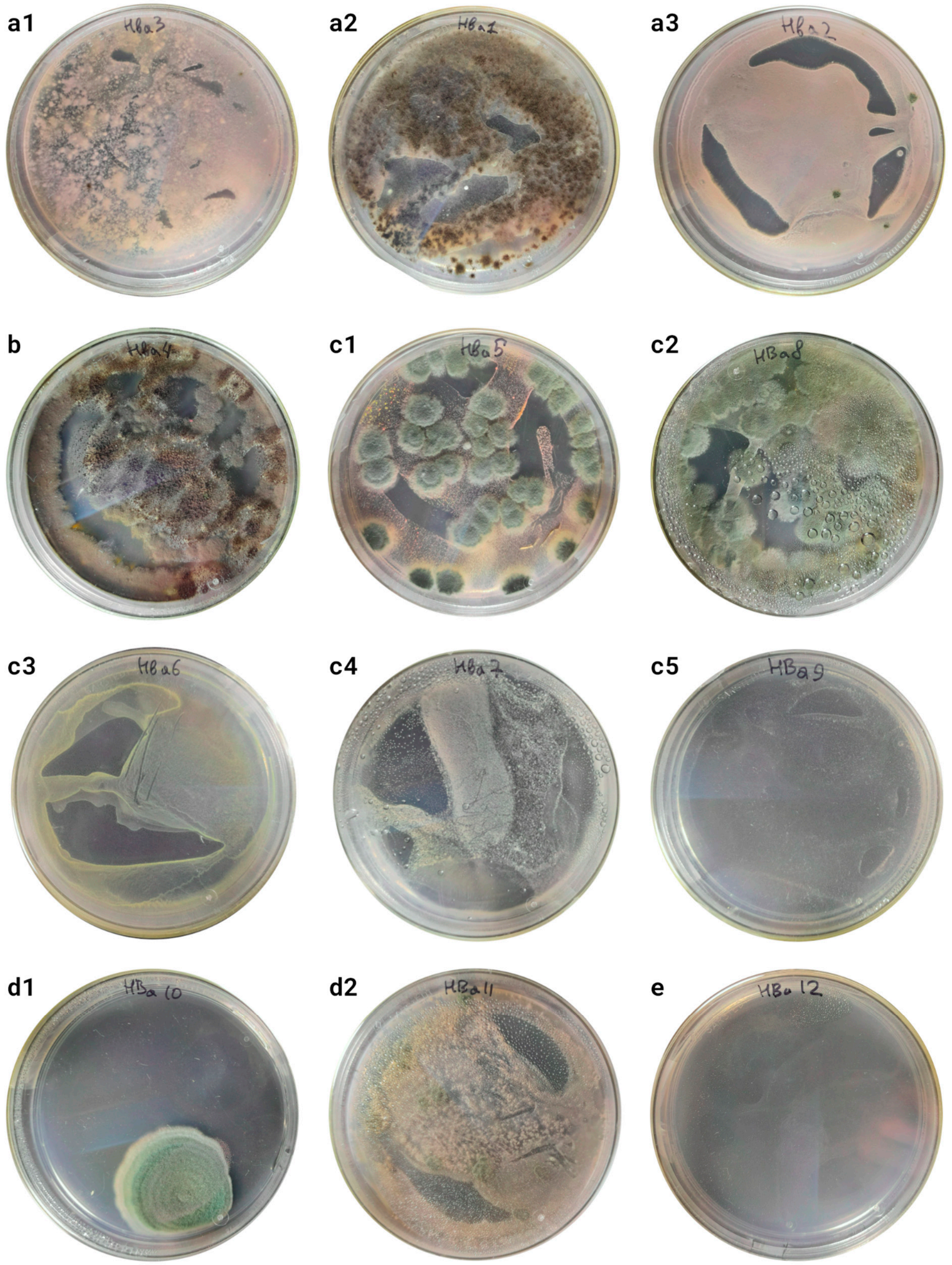

Figure S1. Cultures after enrichment for 3 days on agarose-free Czapek medium, followed by an additional 3 days of cultivation on solid Czapek medium. Extracts from: (a) surfaces of the hive: entrance (a1), wax combs (a2), wooden surface (a3); (b) flushing of container after storaging bee; (c) honey bees with different types of preparations: (c1,c2) the bodies were left in the medium for the entire culturing time; (c3-c5) flushes with sterilized water (c3), physiological solution (c4) and liquid nutrient Czapek medium (c5). (d) Cultures from the surfaces of the 5 dead bees from the hive: (d1) flushes without enrichment cultivated on the solid medium; (d2) the bees were left in a liquid medium similar to c1-c2. (e) Negative control. Diameter of the dish is 9 cm.

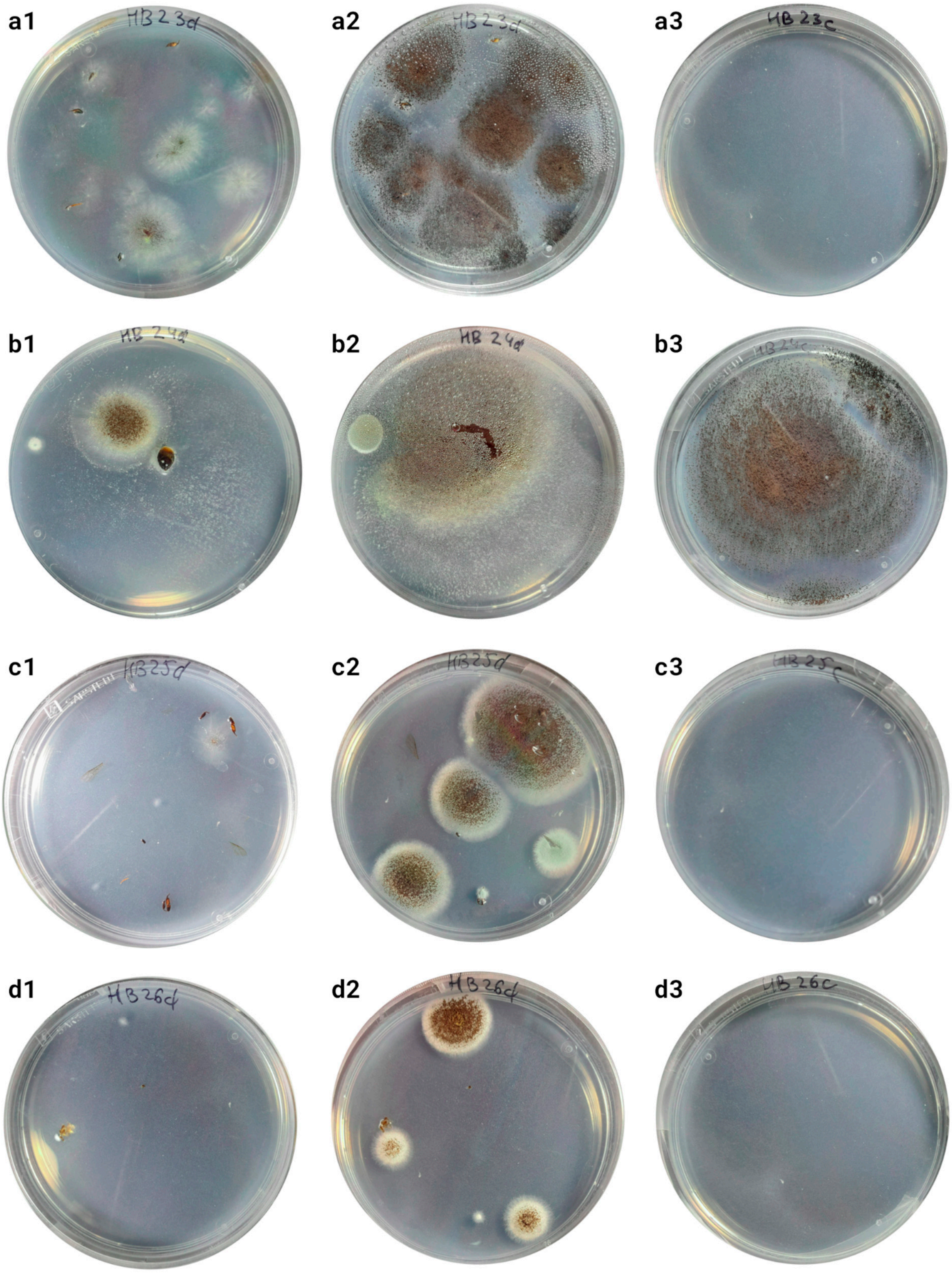

Figure S2. Tests for microbial content on different parts of the bees' body: flushes from limbs and wings with normal saline (a) and with 0.001% SDS added (c); flushes from abdomen with normal saline (b) and with 0.001% SDS added (d). Photographs of culture flushes incubated at 37°C for 12 h before seeding without removal of body parts after three (1) and seven (2) days, and direct flushes without preservation of body parts after seven days (3). Diameter of the dish is 9 cm.

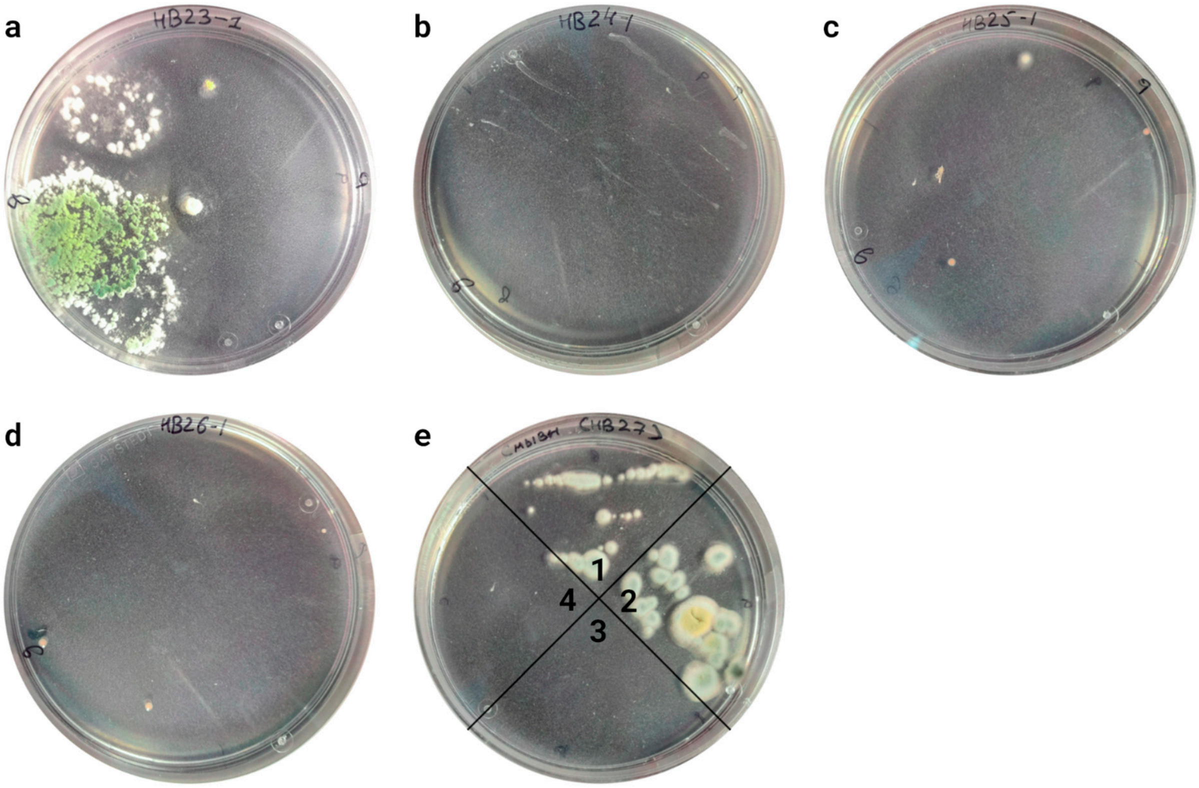

Figure S3. Tests for microbial content on different parts of the bees' body after enrichment for 3 days on agarose-free Czapek medium, followed by an additional 3 days of cultivation on solid Czapek medium: flushes from limbs and wings with normal saline (a) and with 0.001% SDS added (c); flushes from abdomen with normal saline (b) and with 0.001% SDS added (d); from the whole body (e1, e2) and cutted cuticular hairs (e3, e4) without (1,3) and with 0.001% SDS (1,2). Diameter of the dish is 9 cm.

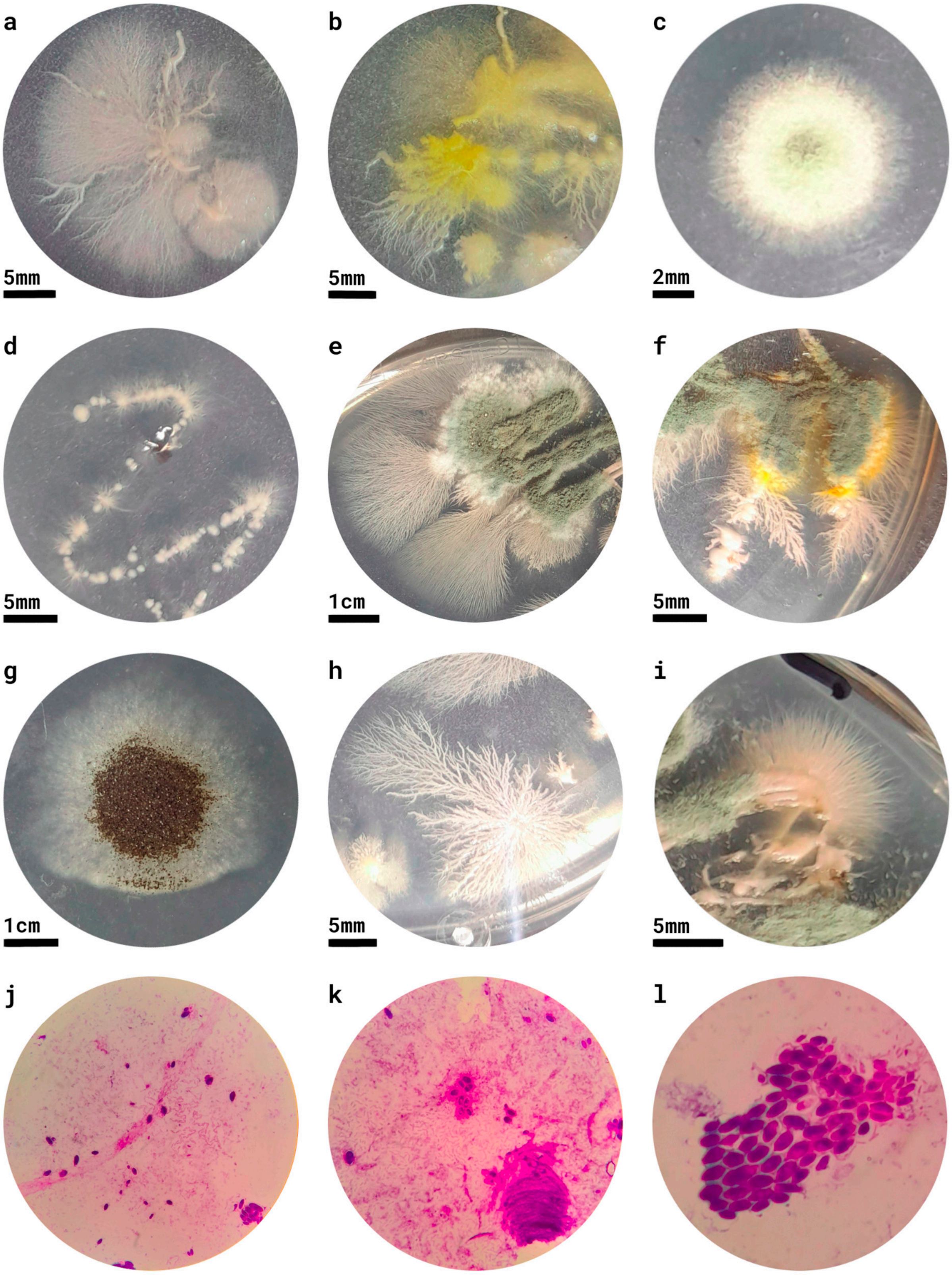

Figure S4. Some morphologies of colonies obtained during culturing of enriched bee surface flushes (a-i) and microscopy results (j-k).

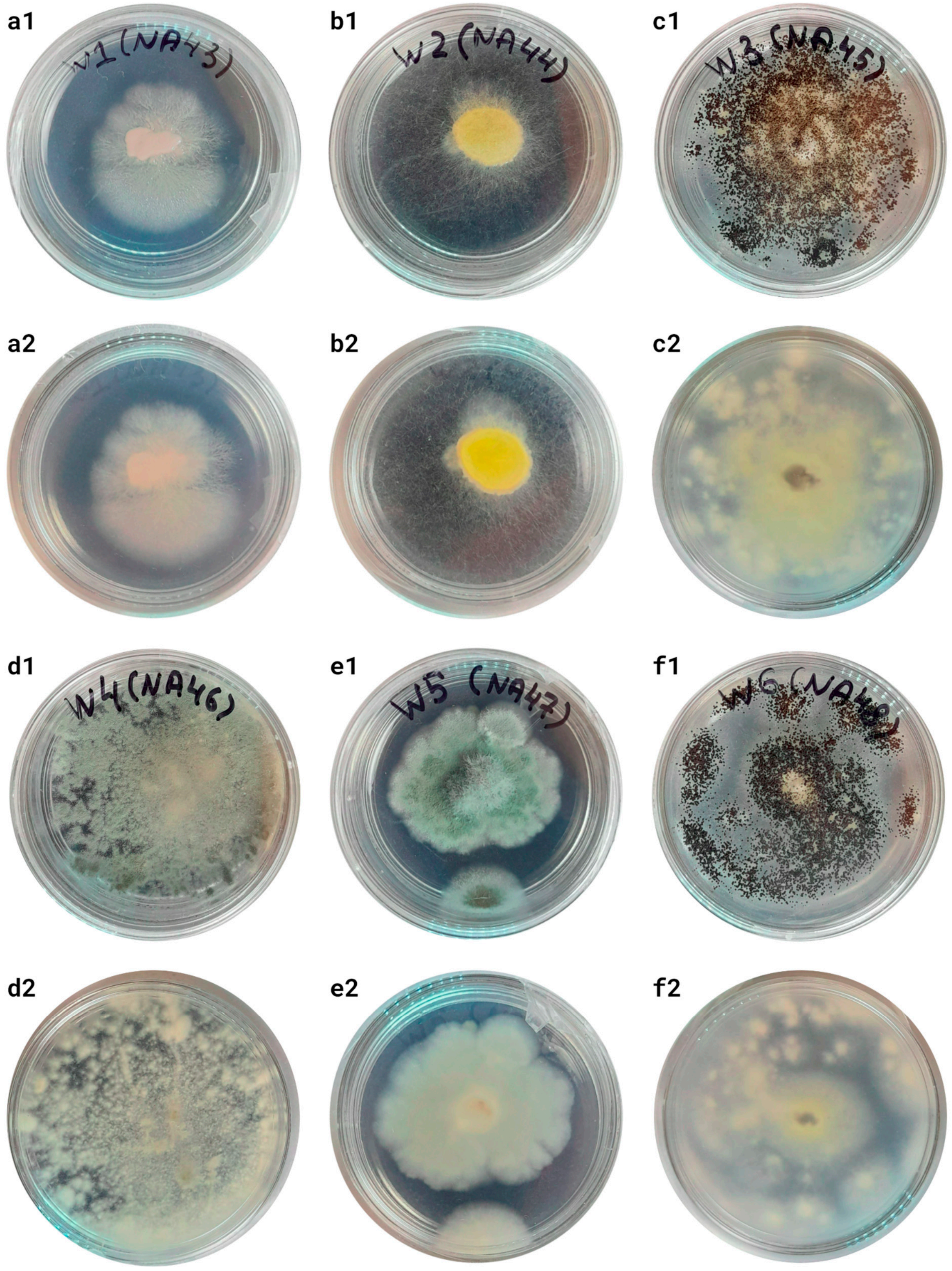

Figure S5. Cultures obtained from the internal hive surfaces (a - e) and individual bee cuticula (f). Same cultures as c-e were also obtained from the surface of bees and in “shattering” tests (for example, cell and cultural morphology of c and f are similar). Petri dishes are photographed from the top (1) and bottom (2). Diameter of the dish is 3 cm. “GRM”-nutrient agar.

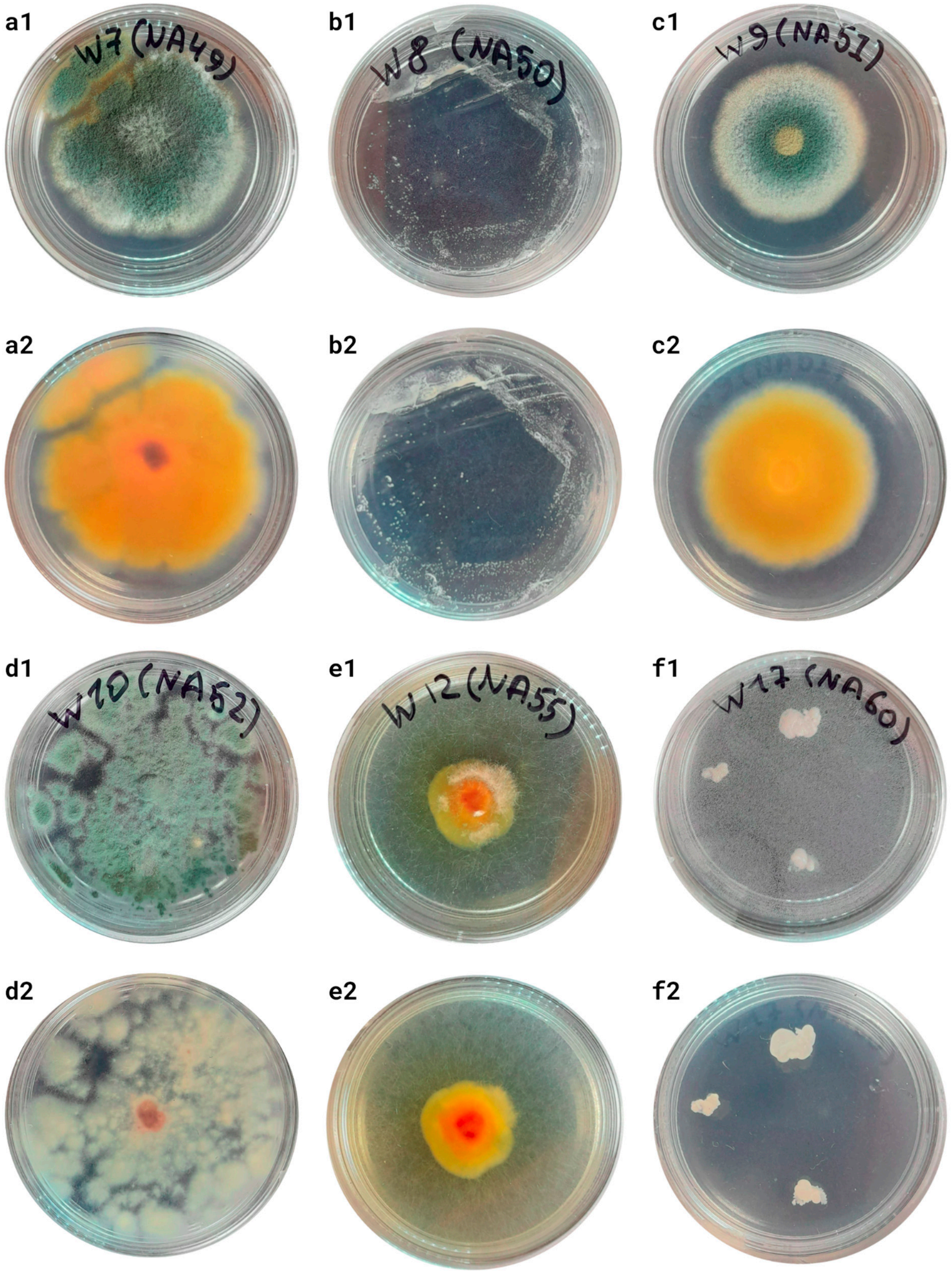

Figure S6. Cultures obtained from the honey bee cuticula. Petri dishes are photographed from the top (1) and bottom (2). Diameter of the dish is 3 cm. “GRM”-nutrient agar.

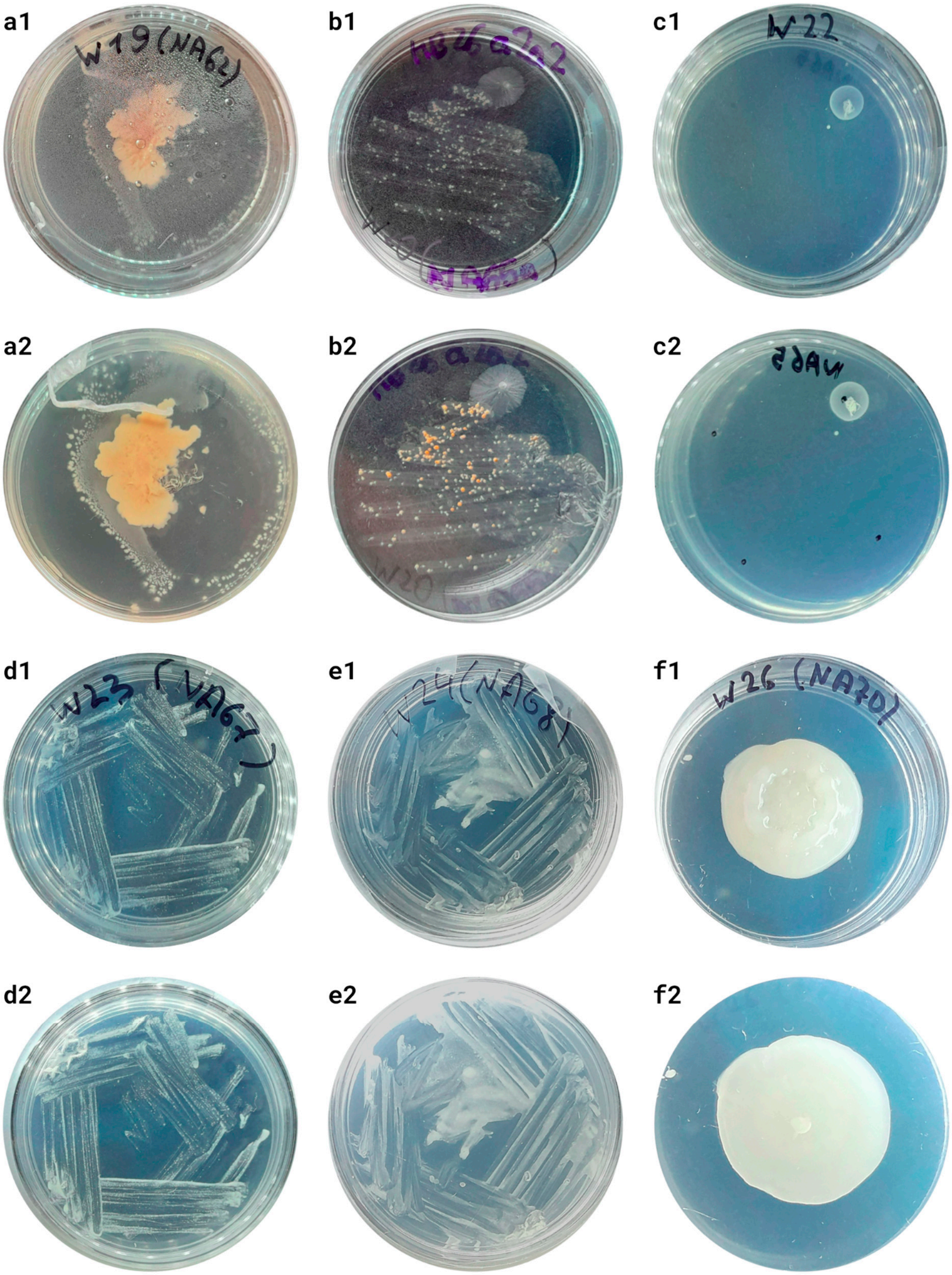

Figure S7. Cultures obtained from the honey bee cuticula. Petri dishes are photographed from the top (1) and bottom (2). Diameter of the dish is 3 cm. “GRM”-nutrient agar.

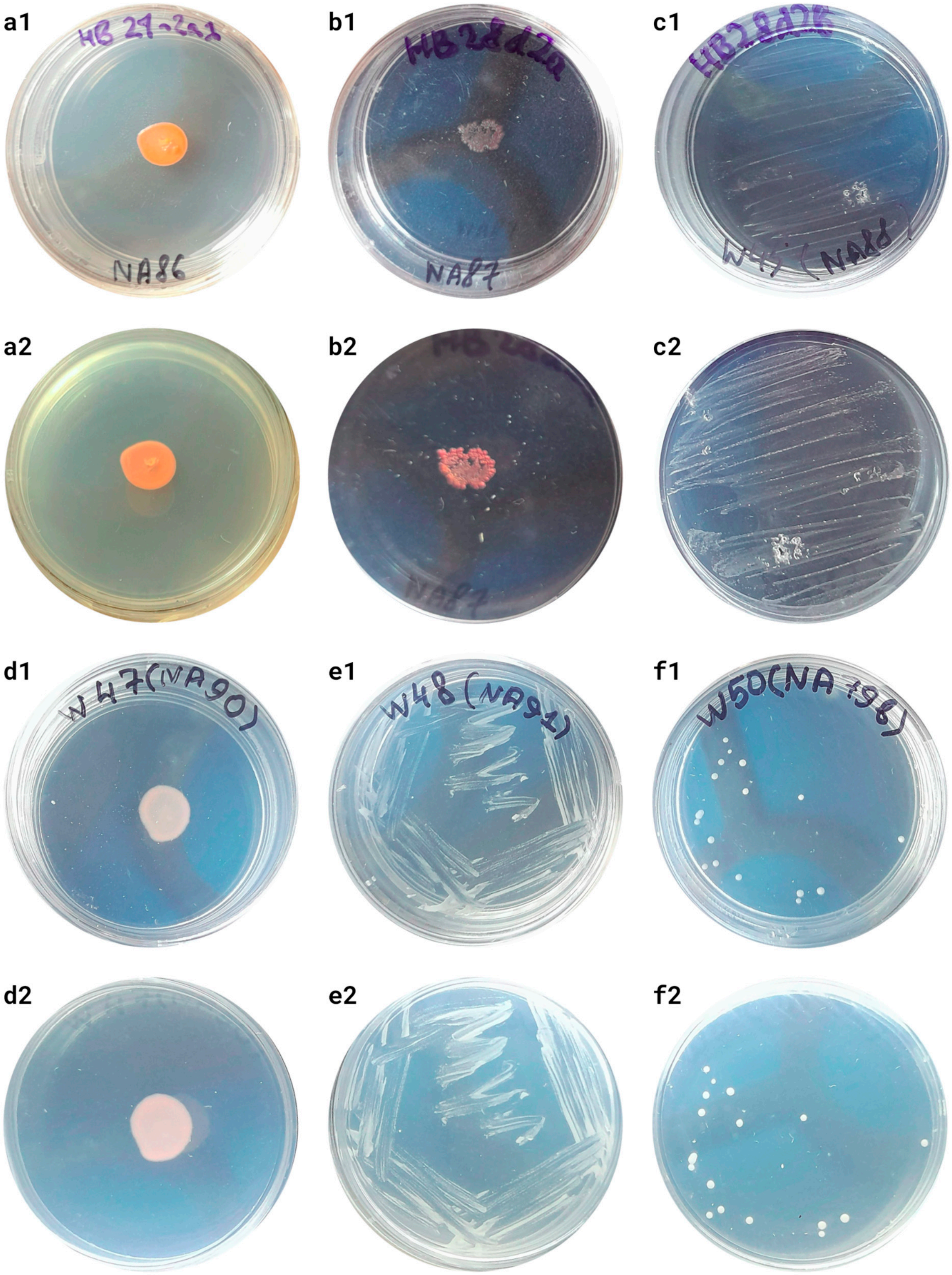

Figure S8. Cultures obtained from the honey bee cuticula. Petri dishes are photographed from the top (1) and bottom (2). Diameter of the dish is 3 cm. “GRM”-nutrient agar.

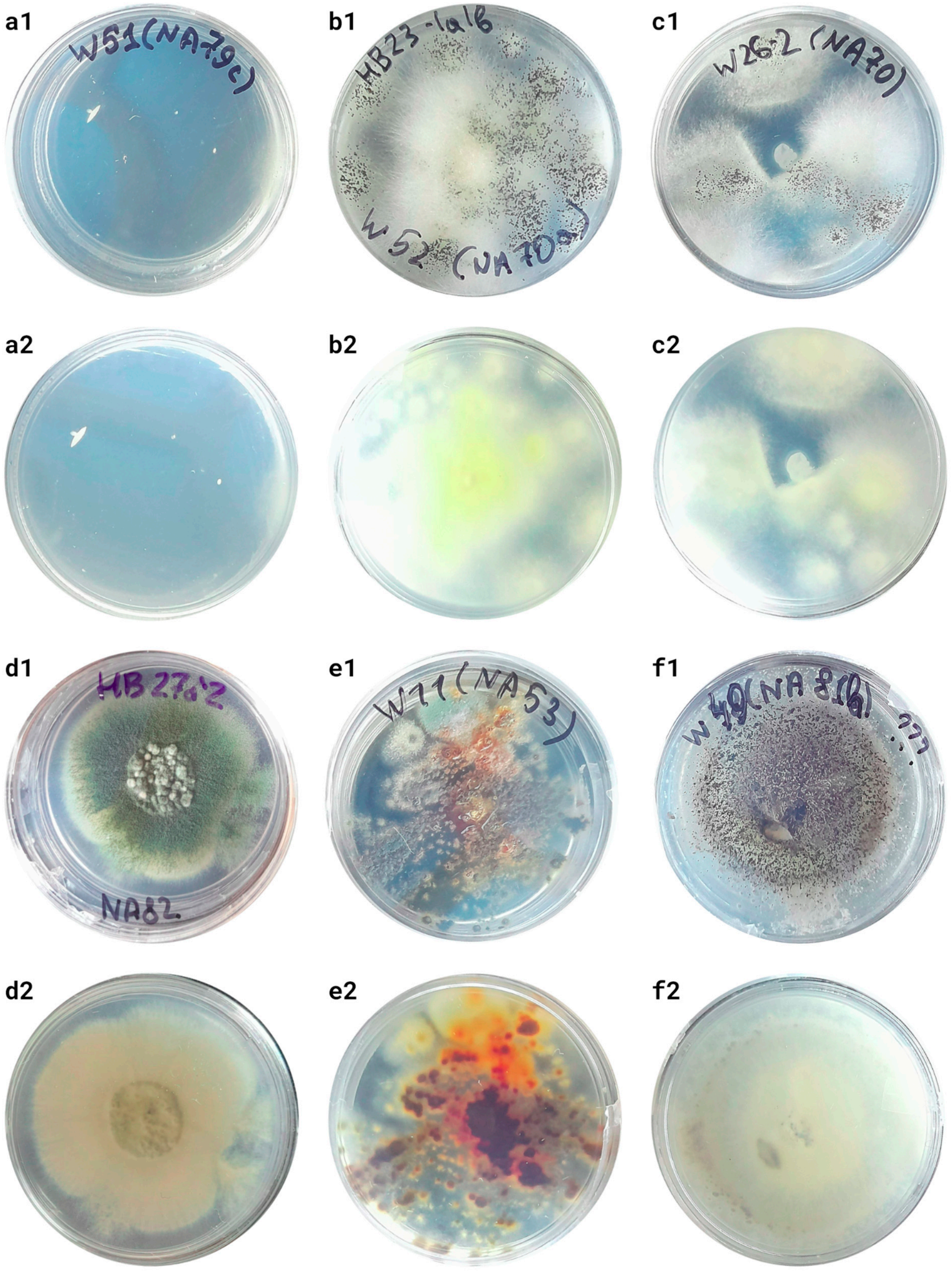

Figure S9. Cultures obtained from the honey bee cuticula. Petri dishes are photographed from the top (1) and bottom (2). Cultures (b) and (c) are similar by colony morphology, but both could be combined cultures. (e) is a combined culture, it is not possible to isolate a pure culture. Diameter of the dish is 3 cm. “GRM”-nutrient agar.

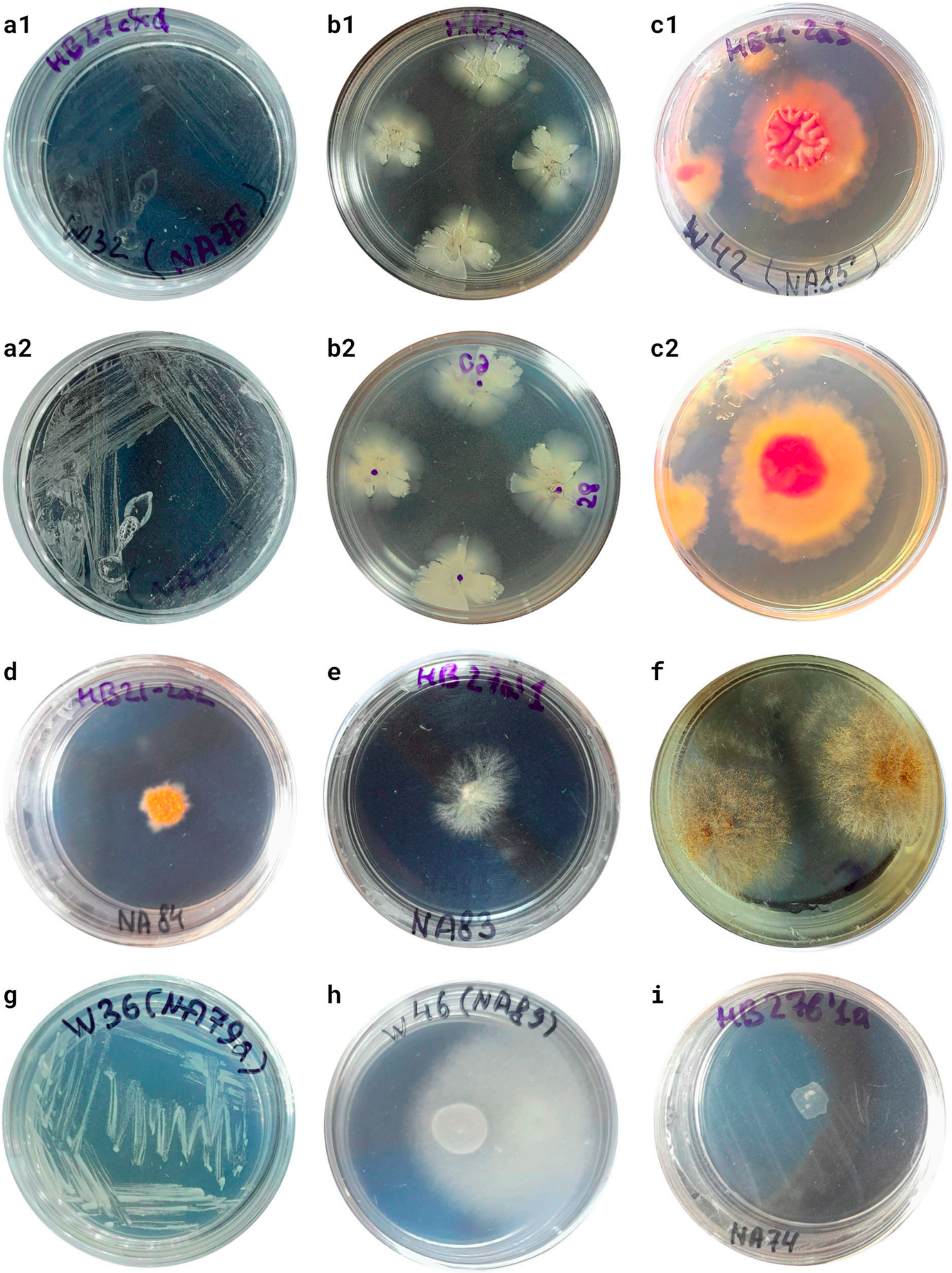

Figure S10. Cultures obtained from the honey bee cuticula. Petri dishes are photographed from the top (1) and bottom (2). Diameter of the dish is 3 cm. “GRM”-nutrient agar.

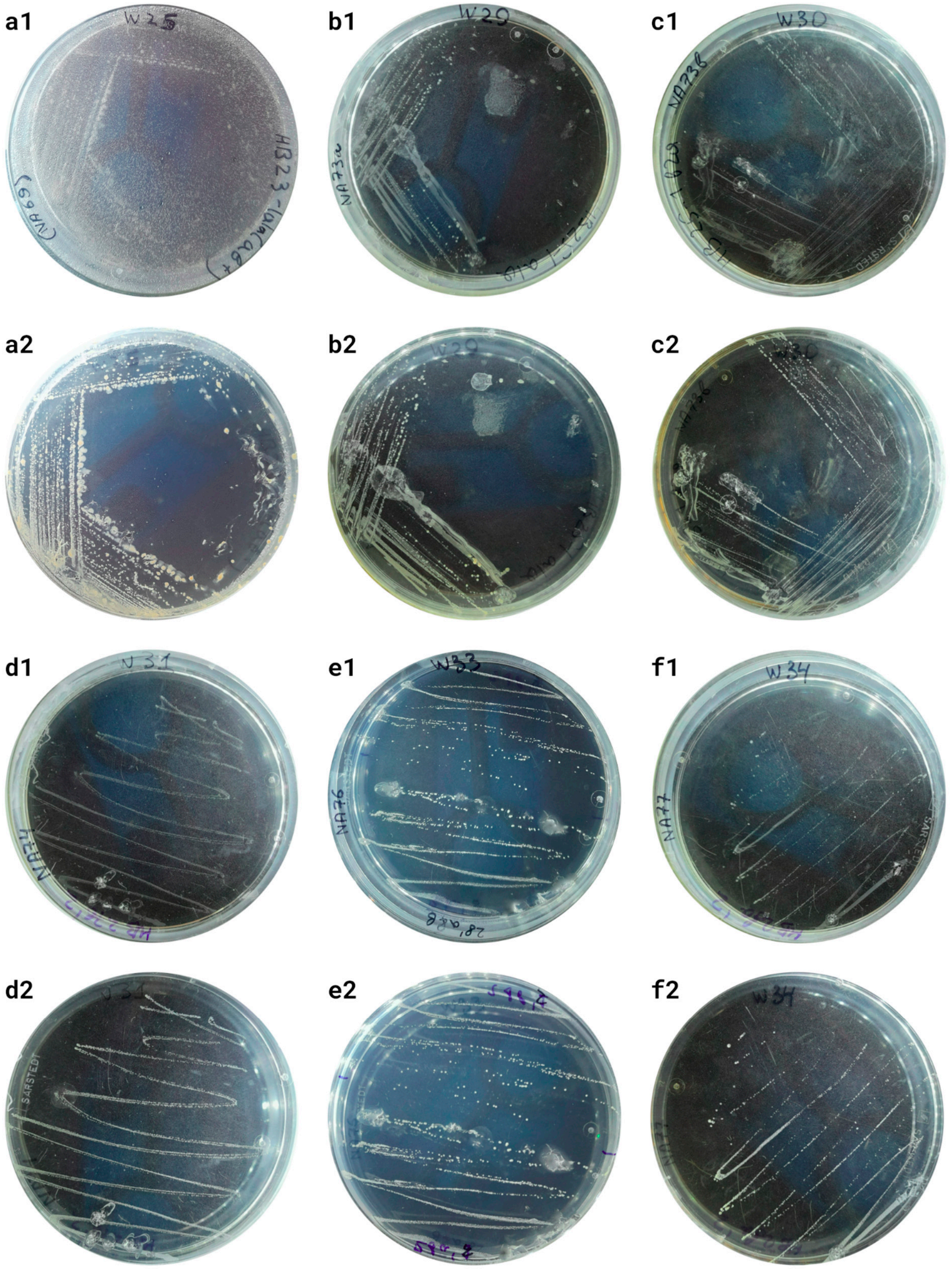

Figure S11. Cultures obtained from the honey bee cuticula. Petri dishes are photographed from the top (1) and bottom (2). Diameter of the dish is 9 cm. "GRM"-nutrient agar.

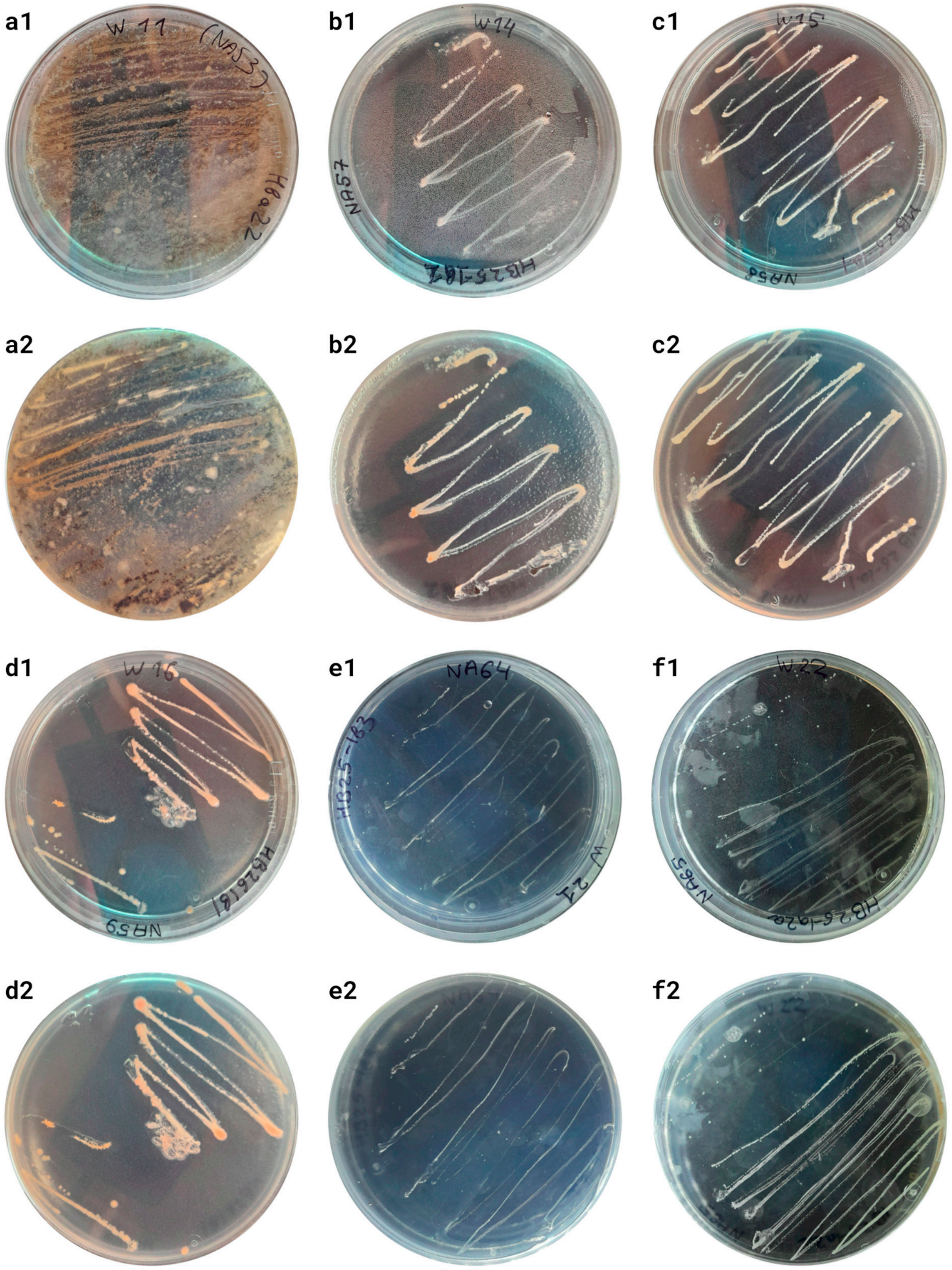

Figure S12. Cultures obtained from the honey bee cuticula. Petri dishes are photographed from the top (1) and bottom (2). (a) could be a combined culture, it is not possible to isolate a pure culture further. Diameter of the dish is 9 cm. “GRM”-nutrient agar.

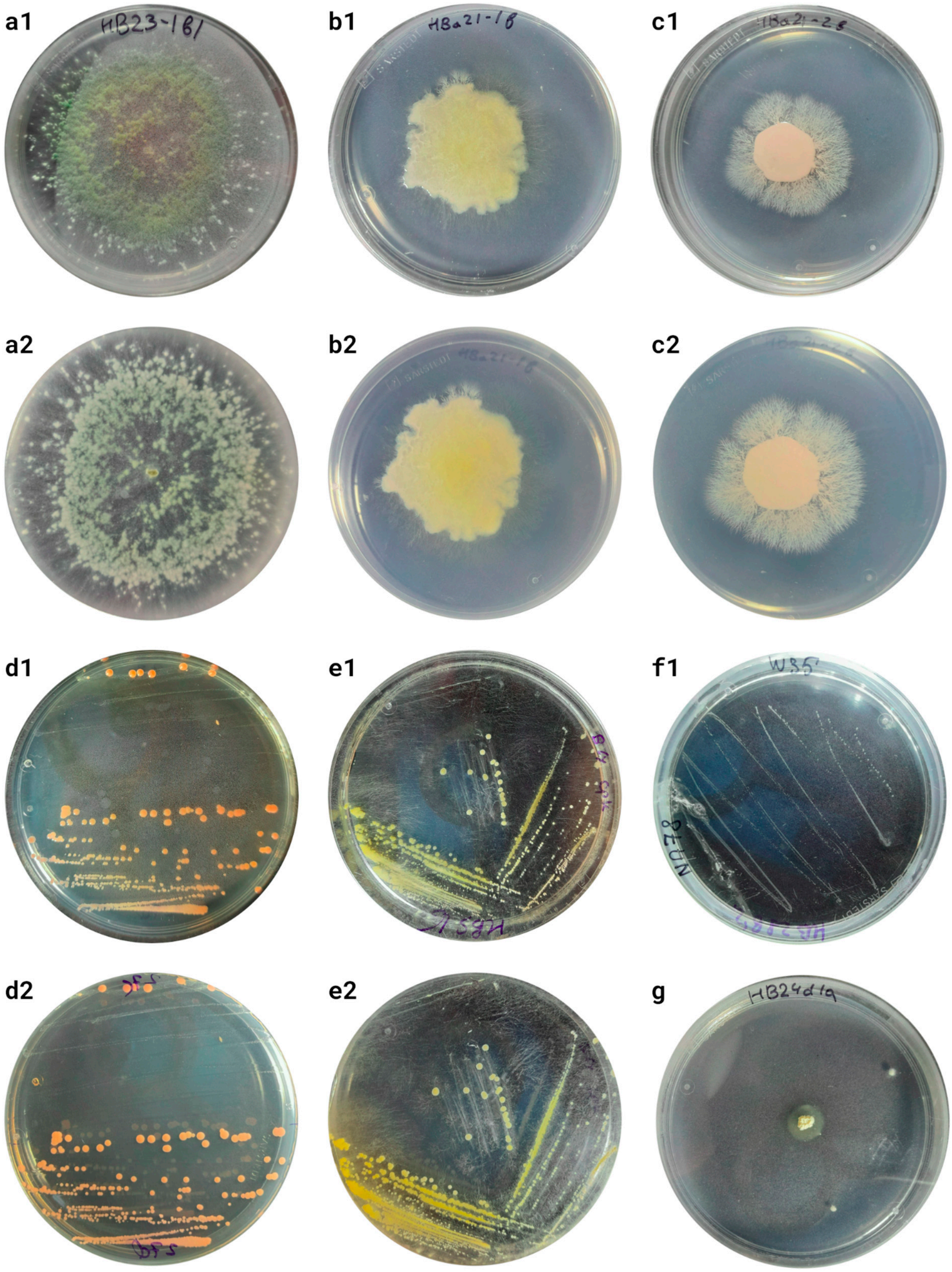

Figure S13. Cultures obtained from the honey bee cuticula. Culture (b) is similar to S5a, and (c) to S5b. Petri dishes are photographed from the top (1) and bottom (2). Diameter of the dish is 9 cm. “GRM”-nutrient agar.

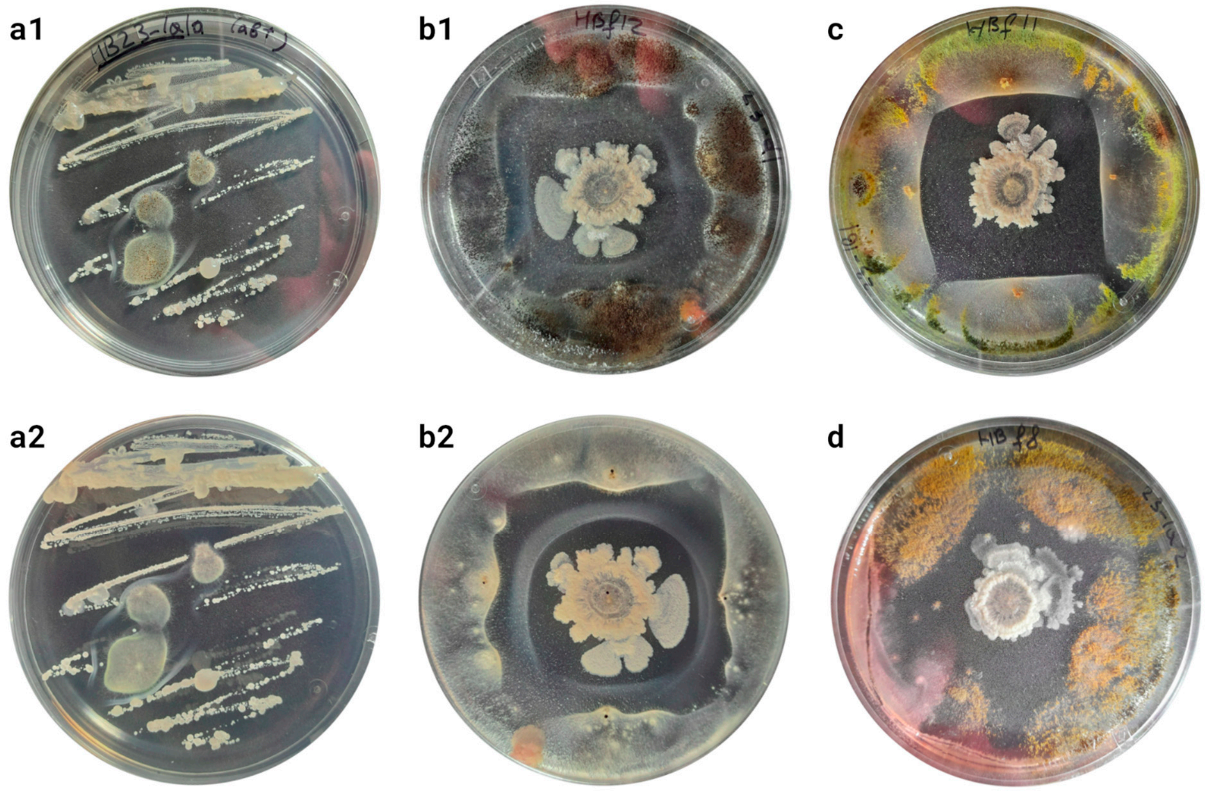

Figure S14. Antibiotic production studies: spot tests. (a) S10b culture which produces antimicrobials. Suppression of S1c (b); S13a (c); S10f (d). A “halo” of unknown nature is observed (b2). Petri dishes are photographed from the top (1) and bottom (2). Diameter of the dish is 9 cm. “GRM”-nutrient agar.

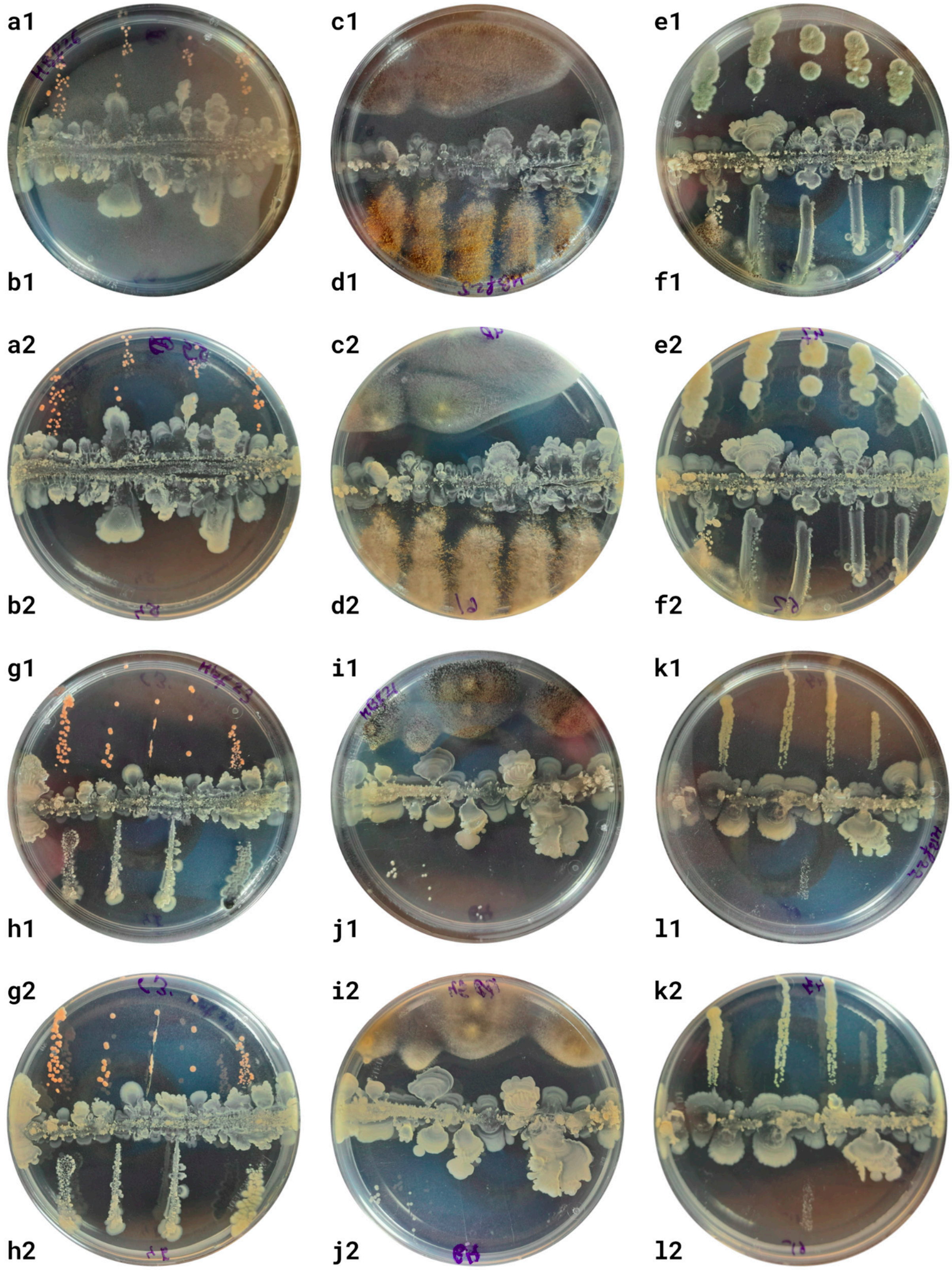

Figure S15. Antibiotics production studies: linear tests. Suppression of (a) S8a (b) S5d (c) S5c (d) S10f (e) S5e (f) S7a (g) S10c (h) S9c (i) S9f (j) S8f (k) S5b (l) S7c. Petri dishes are photographed from the top (1) and bottom (2). The suppression zones are calculated on Figure S16. Diameter of the dish is 9 cm. “GRM”-nutrient agar.

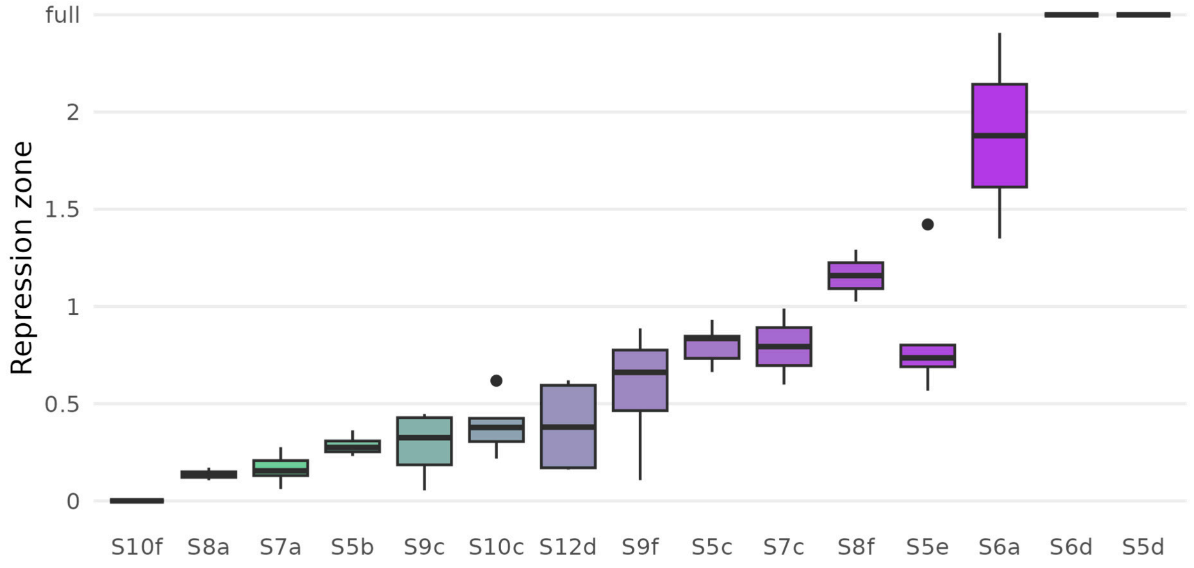

Figure S16. Suppression zones from the linear tests, cm.

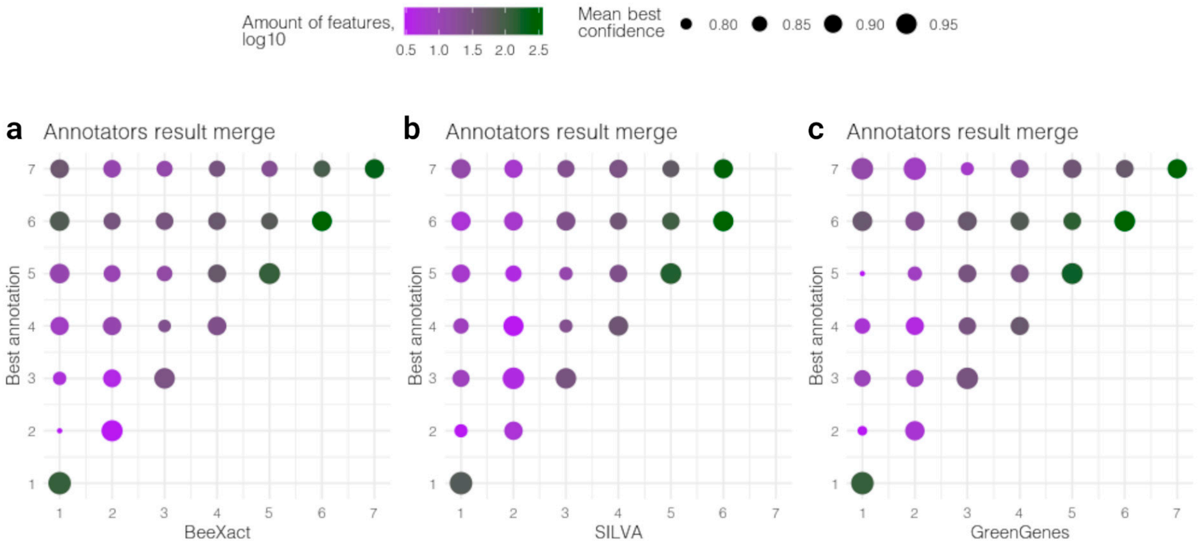

Figure S17. Annotations of 16S rRNA sequencing on different databases. Taxonomy results for different features were merged, and best features were extracted using level and confidence of annotation. (a) BeeXact (best for 526 features). (b) SILVA (best for 409 features). (c) GreenGenes (best for 548 features).

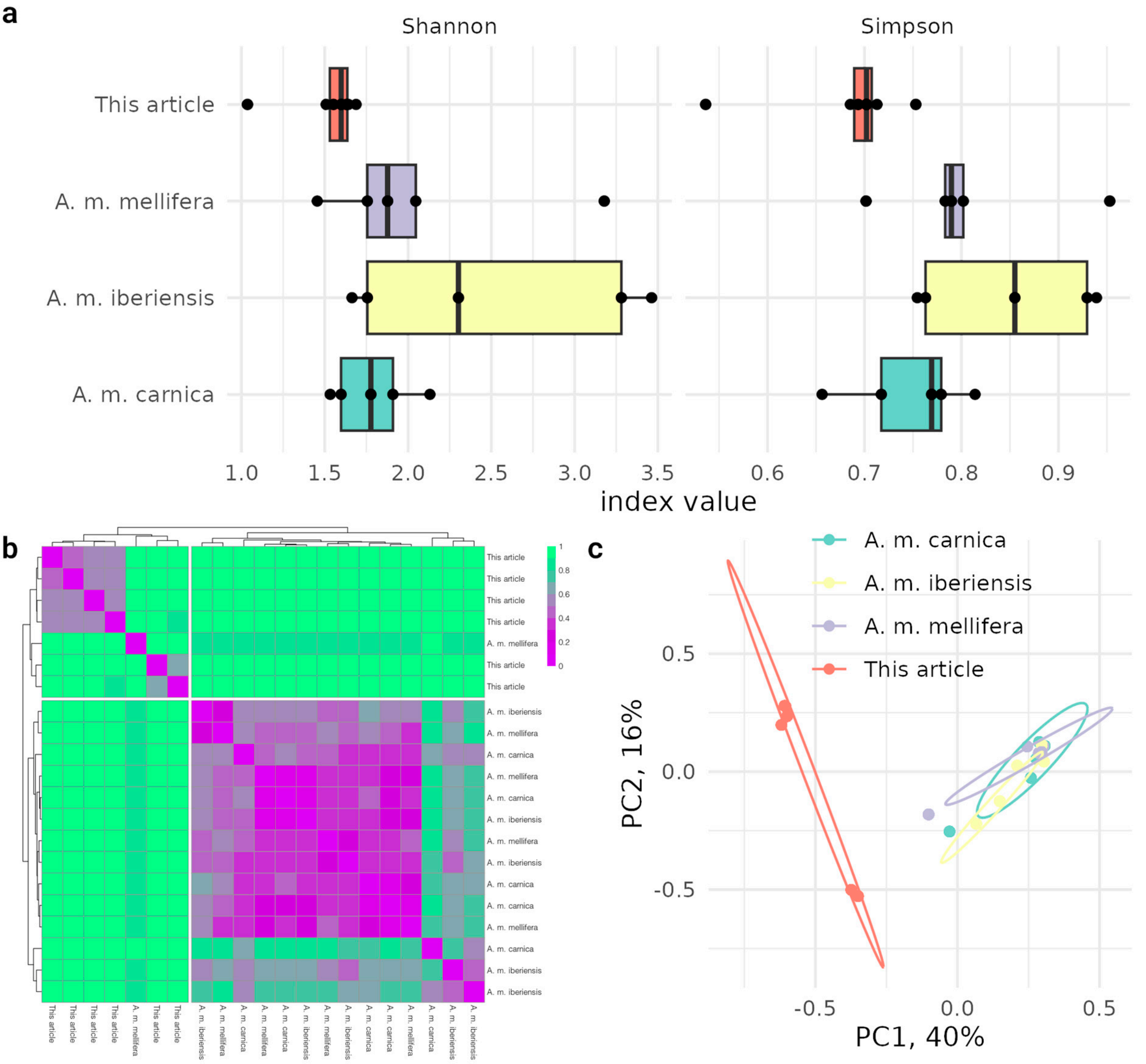

Figure S18. Diversity analysis of bee cuticular pan-metagenome. (a) Alpha-diversity analysis. (b) Heatmap on Bray-Curtis dissimilarity. (c) Beta-PCoA based on Bray-Curtis dissimilarity matrix.

Supplementary table S2. Found genes related to antimicrobial biosynthesis

Product	Genes	Gene count	Common organisms	Chemical class of antibiotic	Antibiotic mechanism of action
Virginiamycin A	vat	1	*Cucumis melo*	AMP and macrolide two component antibiotic	Inhibits protein biosythesis via ribosome 30S subunit binding [1]
Validamycin A	vldW	2	*Streptomyces hygroscopicus, Xanthomonas hortorum*	Oligosaccharide	Inhibits fungal trehelase [2]
Lividomycin	livQ	1	*Streptomyces lividus, Pseudomonas*	Aminoglycoside	Inhibits protein biosythesis via ribosome 30S subunit binding [3]
Penicillin	acyII	1	*Pseudomonas*	Aminopenicilline	Inhibits protein biosythesis via ribosome 30S subunit binding [4]
	ponA	1	*Dictyostelium discoideum, Bacillus subtilis*		
Bacitracin	bcrA	6	*Bacillus licheniformis, Streptococcus faecalis, Thauera aromatica*	AMP	Binds with C55-PP bacterial cell wall precursor with divalent cation participation [5]
	bceA	2	*Bacillus, Burkholderia cepacia*		
	bceB	3	*Bacillus*		
Gramicidin	lgrB	4	*Brevibacillus parabrevis*	AMP	Disrupt bacterial membrane [6]
Colicin V	cvpA	1	*Escherichia coli, Buchnera aphidicola*	AMP	Disrupt bacterial membrane [7]

Novobiocin	novN	1	*Streptomyces niveus*	Aminocoumarin antibiotic	DNA gyrase inhibitor [8]
Streptothricin	satA	1	*Bacillus subtilis*	Aminoglycoside antibiotic	Inhibits protein biosythesis via ribosome 30S subunit binding [9]
Aclacinomycin	rdmC	1	*Streptomyces purpurascens*	Anthracycline antibiotic	DNA or tubulin intercalation [10]
Demethyllacten ocin	tylCV	1	*Streptomyces fradiae*	Macrolide antibiotic	Inhibits protein biosythesis via ribosome 30S subunit binding [11]
Rifampicin	rox	1	*Amycolatopsi s mediterranei, Streptomyces*	Other (small molecule)	Inhibits protein biosynthesis on mRNA synthesis step [12]
Phosphinothrici n	ywnH	1	*Bacillus subtilis, Serratia marcensens*	Other (small molecule)	Binds with glutamine synthetase and invoke ammonia accumulation [13]
Subtilosin	albG	1	*Bacillus subtilis*	AMP	Disrupt bacterial membrane and pore formating [14]
Different toxins	vgrG1	1	*Pseudomonas aeruginosa, Vibrio cholerae*	Proteins	RNase activity and preferentially cleaves at the 3'-end of purine ribonucleotides [15]
	ratA	1	*Rickettsia prowazekii, Shigella flexneri, Escherichia coli*		Binds to 50S ribosomal subunits, preventing them from associating with 30S subunits to form 70S ribosomes and reducing polysome [16]

	relG	1	*Mycobacterium tuberculosis, Microbacterium oxydans*		Has RNase activity and preferentially cleaves at the 3'-end of purine ribonucleotides [17]
	yoeB	1	*Shigella flexneri, Escherichia coli*		Proposed to be an mRNA interferase but also an inhibitor of translation initiation [18]
	mqsR	1	*Escherichia coli*		Plays a role in the control of biofilm formation and induction of persister cells in the presence of antibiotics [19,20]
	fitB	1	*Neisseria gonorrhoeae*		Counteracts the effect of the fitA antitoxin<br>Plays a role in the speed with which bacteria traverse epithelial cells [18]
	higB	3	*Escherichia coli, Shigella boydii*		Counteracts the effect of the HigA antitoxin [21]

Supplementary table S3. Found genes related to antimicrobial resistance

Product	Genes	Gene count
Linearmycin resistance	*lnrL*	7
	lnrN	1
Tunicamycin resistance	*tmrB*	2
Bicyclomycin resistance	*bcr*	9
Fosmidomycin resistance	*fsr*	4
Fosfomycin resistance	*abaF*	5
Bleomycin resistance	*ble*	1
Multidrug resistance proteins	*mdtA*	8
	mdtB	3
	mdtC	2
	mdtD	2
	mdtE	1
	mdtN	1
	mdtH	1
	mdtG	1
	mdtE	1
	norM	4
	ermY	2
	ebrA	1
	yheI	3
	yheH	4

	stp	6
	mexA	1
	mexB	1
	bmr3	7
	bmrA	4
	sepA	3
Quinolone and fluoroquinolones efflux proteins	*abaQ*	2
	norB	1
Tetracycline resistance protein	*tetA*	2
Persistence and stress-resistance toxin	*pasT*	1
Methyl viologen resistance protein	*smvA*	3
Colistin resistance proteins	*emrA*	5
	emrB	5
Microcin C7 self-immunity protein	*mccF*	3
Bifunctional polymyxin resistance protein	*arnA*	1
Antitoxins	*parD*	1
	hicB	1
	higA	2
	mqsA	1

Supplementary references

1. Cocito, C. Antibiotics of the Virginiamycin Family, Inhibitors Which Contain Synergistic Components. *Microbiol. Rev.* **1979**, *43*, 145–192, doi:10.1128/mr.43.2.145-192.1979.
2. Asano, N.; Yamaguchi, T.; Kameda, Y.; Matsui, K. Effect of Validamycins on Glycohydrolases of Rhizoctonia Solani. *J. Antibiot. (Tokyo)* **1987**, *40*, 526–532, doi:10.7164/antibiotics.40.526.
3. Yamaguchi, M.; Eda, J.; Kobayashi, F.; Mitsuhashi, S. Mode of Action of Lividomycin on Protein Synthesis in *Escherichia Coli*. *Antimicrob. Agents Chemother.* **1973**, *4*, 380–382, doi:10.1128/AAC.4.3.380.
4. Lederberg, J. MECHANISM OF ACTION OF PENICILLIN. *J. Bacteriol.* **1957**, *73*, 144–144, doi:10.1128/jb.73.1.144-144.1957.
5. Siewert, G.; Strominger, J.L. BACITRACIN: AN INHIBITOR OF THE DEPHOSPHORYLATION OF LIPID PYROPHOSPHATE, AN INTERMEDIATE IN THE BIOSYNTHESIS OF THE PEPTIDOGLYCAN OF BACTERIAL CELL WALLS. *Proc. Natl. Acad. Sci.* **1967**, *57*, 767–773, doi:10.1073/pnas.57.3.767.
6. Yonezawa, H.; Okamoto, K.; Tomokiyo, K.; Izumiya, N. Mode of Antibacterial Action by Gramicidin S. *J. Biochem. (Tokyo)* **1986**, *100*, 1253–1259, doi:10.1093/oxfordjournals.jbchem.a121831.
7. Yang, C.C.; Konisky, J. Colicin V-Treated Escherichia Coli Does Not Generate Membrane Potential. *J. Bacteriol.* **1984**, *158*, 757–759, doi:10.1128/jb.158.2.757-759.1984.
8. Bisacchi, G.S.; Manchester, J.I. A New-Class Antibacterial—Almost. Lessons in Drug Discovery and Development: A Critical Analysis of More than 50 Years of Effort toward ATPase Inhibitors of DNA Gyrase and Topoisomerase IV. *ACS Infect. Dis.* **2015**, *1*, 4–41, doi:10.1021/id500013t.
9. Morgan, C.E.; Kang, Y.-S.; Green, A.B.; Smith, K.P.; Dowgiallo, M.G.; Miller, B.C.; Chiaraviglio, L.; Truelson, K.A.; Zulauf, K.E.; Rodriguez, S.; et al. Streptothricin F Is a Bactericidal Antibiotic Effective against Highly Drug-Resistant Gram-Negative Bacteria That Interacts with the 30S Subunit of the 70S Ribosome. *PLOS Biol.* **2023**, *21*, e3002091, doi:10.1371/journal.pbio.3002091.
10. Misumi, M.; Yamaki, H.; Akiyama, T.; Tanaka, N. Mechanism of Action of Aclacinomycin A. II. The Interaction with DNA and with Tubulin. *J. Antibiot. (Tokyo)* **1979**, *32*, 48–52, doi:10.7164/antibiotics.32.48.
11. Baltz, R.H.; Seno, E.T.; Stonesifer, J.; Wild, G.M. Biosynthesis of the Macrolide Antibiotic Tylosin. A Preferred Pathway from Tylactone to Tylosin. *J. Antibiot. (Tokyo)* **1983**, *36*, 131–141, doi:10.7164/antibiotics.36.131.
12. Campbell, E.A.; Korzheva, N.; Mustaev, A.; Murakami, K.; Nair, S.; Goldfarb, A.; Darst, S.A. Structural Mechanism for Rifampicin Inhibition of Bacterial RNA Polymerase. *Cell* **2001**, *104*, 901–912, doi:10.1016/S0092-8674(01)00286-0.
13. Forlani, G.; Obojska, A.; Berlicki, Ł.; Kafarski, P. Phosphinothricin Analogues as Inhibitors of Plant Glutamine Synthetases. *J. Agric. Food Chem.* **2006**, *54*, 796–802, doi:10.1021/jf0526285.
14. Sutyak Noll, K.; Sinko, P.J.; Chikindas, M.L. Elucidation of the Molecular Mechanisms of Action of the Natural Antimicrobial Peptide Subtilosin Against the Bacterial Vaginosis-Associated Pathogen Gardnerella Vaginalis. *Probiotics Antimicrob. Proteins* **2011**, *3*, 41–47, doi:10.1007/s12602-010-9061-4.
15. Ma, A.T.; Mekalanos, J.J. In Vivo Actin Cross-Linking Induced by *Vibrio Cholerae* Type VI Secretion System Is Associated with Intestinal Inflammation. *Proc. Natl. Acad. Sci.* **2010**, *107*, 4365–4370, doi:10.1073/pnas.0915156107.
16. Zhang, Y.; Inouye, M. RatA (YfjG), an *Escherichia Coli* Toxin, Inhibits 70S Ribosome

Association to Block Translation Initiation. *Mol. Microbiol.* **2011**, *79*, 1418–1429, doi:10.1111/j.1365-2958.2010.07506.x.

17. Ramage, H.R.; Connolly, L.E.; Cox, J.S. Comprehensive Functional Analysis of Mycobacterium Tuberculosis Toxin-Antitoxin Systems: Implications for Pathogenesis, Stress Responses, and Evolution. *PLoS Genet.* **2009**, *5*, e1000767, doi:10.1371/journal.pgen.1000767.
18. Nieto, C.; Cherny, I.; Khoo, S.K.; De Lacoba, M.G.; Chan, W.T.; Yeo, C.C.; Gazit, E.; Espinosa, M. The *yefM-yoeB* Toxin-Antitoxin Systems of *Escherichia Coli* and *Streptococcus Pneumoniae* : Functional and Structural Correlation. *J. Bacteriol.* **2007**, *189*, 1266–1278, doi:10.1128/JB.01130-06.
19. De Souza-Neto, R.R.; Carvalho, I.G.B.; Martins, P.M.M.; Picchi, S.C.; Tomaz, J.P.; Caserta, R.; Takita, M.A.; De Souza, A.A. MqsR Toxin as a Biotechnological Tool for Plant Pathogen Bacterial Control. *Sci. Rep.* **2022**, *12*, 2794, doi:10.1038/s41598-022-06690-x.
20. Yamaguchi, Y.; Park, J.-H.; Inouye, M. MqsR, a Crucial Regulator for Quorum Sensing and Biofilm Formation, Is a GCU-Specific mRNA Interferase in Escherichia Coli. *J. Biol. Chem.* **2009**, *284*, 28746–28753, doi:10.1074/jbc.M109.032904.
21. Xu, B.-S.; Liu, M.; Zhou, K.; Geng, Z.; Gao, Z.-Q.; Dong, Y.-H.; She, Z.; Liu, Q.-S. Conformational Changes of Antitoxin HigA from Escherichia Coli Str. K-12 upon Binding of Its Cognate Toxin HigB Reveal a New Regulation Mechanism in Toxin-Antitoxin Systems. *Biochem. Biophys. Res. Commun.* **2019**, *514*, 37–43, doi:10.1016/j.bbrc.2019.04.061.
